# New Concept in Bioderived Composites: Biochar as Toughening Agent for Improving Performances and Durability of Agave-Based Epoxy Biocomposites

**DOI:** 10.3390/polym13020198

**Published:** 2021-01-08

**Authors:** Bernardo Zuccarello, Mattia Bartoli, Francesco Bongiorno, Carmelo Militello, Alberto Tagliaferro, Antonio Pantano

**Affiliations:** 1 Dipartimento di Ingegneria, Università degli Studi di Palermo, 90128 Palermo, Italy; bernardo.zuccarello@unipa.it (B.Z.); francesco.bongiorno01@unipa.it (F.B.); carmelo.militello01@unipa.it (C.M.); antonio.pantano@unipa.it (A.P.); 2 Department of Applied Science and Technology (DISAT), Politecnico di Torino, 10129 Torino, Italy; mattia.bartoli@polito.it

**Keywords:** agave, biochar, thermoset composites, fatigue

## Abstract

Biocomposites are increasingly used in the industry for the replacement of synthetic materials, thanks to their good mechanical properties, being lightweight, and having low cost. Unfortunately, in several potential fields of structural application their static strength and fatigue life are not high enough. For this reason, several chemical treatments on the fibers have been proposed in literature, although still without fully satisfactory results. To overcome this drawback, in this study we present a procedure based on the addition of a carbonaceous filler to a green epoxy matrix reinforced by Agave sisalana fibers. Among all carbon-based materials, biochar was selected for its environmental friendliness, along with its ability to improve the mechanical properties of polymers. Different percentages of biochar, 1, 2, and 4 wt %, were finely dispersed into the resin using a mixer and a sonicator, then a compression molding process coupled with an optimized thermomechanical cure process was used to produce a short fiber biocomposite with Vf = 35%. Systematic experimental tests have shown that the presence of biochar, in the amount 2 wt %, has significant effects on the matrix and fiber interphase, and leads to an increase of up to three orders of magnitude in the fatigue life, together with an appreciable improvement in static tensile strength.

## 1. Introduction

The use of composite materials reinforced by natural fibers is growing strongly in many industrial fields, particularly in the automotive sector [1,2,3], but also in the civil construction area [2] and in naval production [3]. German carmakers, soon followed by other manufacturers, took the lead in introducing natural fiber composites for interior and exterior applications: door panels, parcel shelves, seat cushions, dashboard parts, backrests, mirror casing, projector cover, voltage stabilizer cover, helmet, roof linings, etc. In the civil construction area they can be used for: beams, building panels, roofing products, autoclaved cement composite, and water tanks. For shipbuilding, the adoption of green composites can potentially represent a valid substitute for fiberglass. These include both purposely grown and harvested fibers, as well as those recovered from agricultural waste. Thanks to their recyclability and renewability, biocomposites allow to comply with more and more stringent environmental protection regulations [4,5] improving also the cost effectiveness [6]. Increasing the mechanical performance of these materials is a mandatory task to spread their use not only in non-structural applications, but also in semi- and proper structural applications actually limited by their failure mechanisms [7]. The experimental evidence has shown that biocomposites reinforced by natural fibers present damage mechanisms [8,9,10,11,12,13] somewhat quite similar to those observed in traditional composites reinforced by synthetic fibers [14], as debonding, delamination, inter-fiber fractures and fiber fractures. When fiber-reinforced polymers are tested with axial or multiaxial tensile loading, the damage mechanisms happen in a particular order [15]. In a first phase matrix, cracks in-between the reinforcing fibers in off-axis layer form and grow. The second phase is characterized by a saturation of transverse matrix cracks, transversal tensile failure and longitudinal inter fiber fractures. Degradation progresses slowly before phase three begins and fiber-reinforced polymer rapidly collapses due to the growth of delamination and fiber fractures. The depletion of this behaviour is the great challenge of bioreinforced plastics and the addition of strengthening additives is the most effective way to avoid it. Several high-tech carbonaceous fillers (i.e., graphene oxides [16], carbon nanotubes [17]) have been used but their performances are counterbalanced by the high-cost and environmental unfriendly production [18,19]. A game-change event could be represented by the use of biochar produced from biomass waste streams through pyrolysis [20]. As demonstrated by Woolf and co-workers [21], the implementation of biochar as soil additive contribute to the reduction in green-house gases emissions together, but biochar is far more than a mere soil amendment [22]. It is a multifunctional platform for material science [23], as Mohanty and co-workers [24] clearly demonstrated for the production of performing composites. In the very same field, biochar has been proved to be a surprising and versatile filler able to improve the mechanical [25,26], thermal [27,28,29] and electrical [30,31,32] properties of plenty of the polymeric matrix. The strength of biochar is the high productive flexibility that improves its cost-effectiveness balancing the material properties with biomass streams availability.

Bartoli et al. [33] showed how the biomass used affected the biochar performances of epoxy matrix at very low concentration of 2 wt % with a selective magnification of Young’s modulus or elongation. While soil amendment biochar has far to go in order to contribute to the mitigation of the humankind environmental impact [34], as high valued filler in material science [35,36,37] biochar could play a major role in the green economy transition [38,39]. Additionally, Matykiewicz [40] showed the beneficial effect of biochar as toughening agent in carbon fibers based epoxy composites.

In the present work, the effect of biochar produced from coffee waste stream was used as additive to epoxy biocomposites reinforced by *Agave sisalana* fibers (sisal). This fibrous materials combines remarkable mechanical properties together with a 75% green-house gas emissions compared with glass fibers [41]. Related composites containing short-fiber with random distribution appreciated for their good stiffness. Nonetheless, they are characterized by low resistance due to the peculiar “transversal” damage processes strictly influenced by debonding and pull-out phenomena related to fiber-matrix adhesion. Considering the positive effects of biochar on the mechanical properties of polymer matrix composites, the present work analyses its effects on the fatigue performance of biocomposites reinforced by sisal obtained with a compression molding process.

Our aim is to produce a bioderived based high-performance polymer with an improved life durability for contribute to the mitigation of reinforced plastic effect on environment for applications in the automotive [42], civil construction [2,43], and naval [3] sectors.

## 2. Materials and Methods

### 2.1. Materials

For the realization of the biocomposites, the fibers were opportunely extracted from mature leaves of agave sisalana. The selection process of the structural fibers, already optimized in previous works [8,9,10,11,12,13], consists in selecting only the perimeter fibers from the middle third of mature leaves (4–5 years), discarding the less resistant non-structural central fibers (see [11] for more details).

In brief, the selected fibers have a typical horseshoe transversal section with a mean diameter that fall in the range 100 to 250 μm, value that is in practice about 10 times greater than that of the common synthetical fibers used in the Polymer Matrix Composites (PMCs). As all the agave fibers, they contain characteristic sub-fibers, having a diameter between 10 and 30 μm, with walls made by hemicellulose and lignin reinforced by cellulose spirals having winding angle of about 20°; also, their composition falls in the ranges typically reported in literature for the sisal fibers (40–88% lignin, 8–24% cellulose, 2–28% hemicellulose). The specific weight is about 14.4 kN/m^3^ (significantly lower than that of the synthetical fibers) and the main tensile properties, obtained by single fiber tensile test, are: Young modulus *E* ≈ 40 GPa and tensile strength *σ_R_* = 690 MPa.

Once selected, the fresh fibers (without any pre-treatment) were cut with an optimal length of 4 ± 2 mm [44] and mixed with a green epoxy matrix produced by the American Entropy Resin Inc. (San Antonio, CA, USA), named SUPERSAP CNR, with IHN-type hardener. The SUPERSAP CNR epoxy resin is produced by using an ecofriendly manufacturing process through green chemistry, sustainable raw materials, and efficient manufacturing conserving energy, minimizing harmful byproducts and reducing greenhouse gas emissions of resins and hardeners. Using Life Cycle Assessment (LCA), the producer has demonstrated how SUPERSAP CNR reduce the environmental impact of the products [45]. Preliminary tensile tests have shown that such a matrix exhibits a tensile strength of about 35 MPa at a failure strain of about 2%. In order to toughen the matrix, as well as to obtain possible bridging-effects between fibers or between matrix and fibers, and evaluate the possible increase in static and fatigue strength of the biocomposites under study, biochar have been finely dispersed within the matrix using initially a mixer and then a FLOUREON sonicator (having a power of 50 W and a frequency of 40 KHz) for 30 min at a temperature of 30–35 °C, with subsequent cooling phase of the mixture for about 10 min, and final mixing of the hardener. The biochar used was produced using a pilot-scale rotary kiln pyrolysis unit by UK Biochar research center [46] setting the highest treatment temperature (HTT) to 550 °C with a kiln residence time of 15 min and a HTT residence of 4 min.

Biochar was analyzed by using several techniques to establish several key surface and morphological properties.

Infrared spectroscopy was performed using a Fourier transformed infrared (FT-IR) spectrometer (Nicolet 5700, Thermoscientific, Waltham, MA, USA) operated in attenuated total reflectance (ATR) equipped with a diamond window (Smartorbit, Thermoscientific, Waltham, MA, USA).

Raman spectroscopy was performed using Renishaw^®^ Ramanscope InVia (H43662 model, Gloucestershire, UK).

The particle size distribution was evaluated using a laser granulometry (Fritsch Analysette 22, Idar–Oberstein, Germany) after dispersion in ethanol and sonication in an ultrasonic bath for 10 min.

### 2.2. Processing

**Figure 1:**
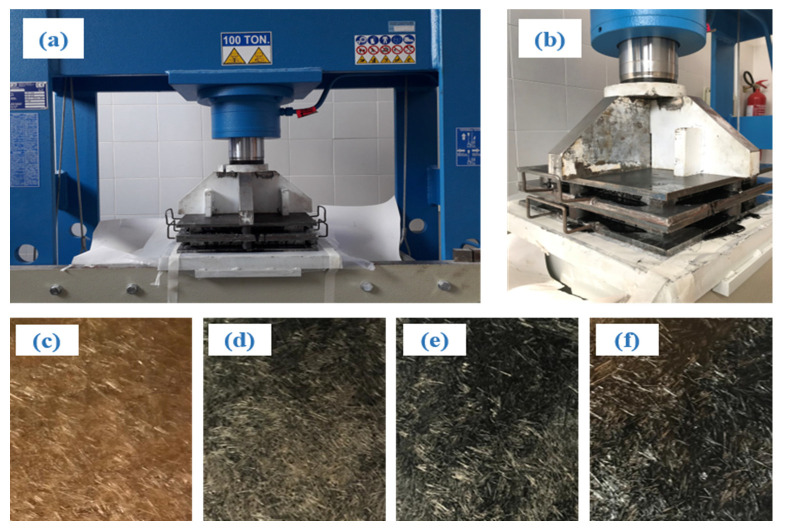
(**a**) Mould, counter-mould and hydraulic press, (**b**) final phase of the compression molding process (note the excess resin leakage), (**c**) panel of R+A, (**d**) BC1%, (**e**) BC2%, and (**f**) BC4% biocomposite.

Preliminary studies carried out on polymeric matrix (PLA, epoxy resin) biocomposites reinforced by agave fibers with different orientation (random, unidirectional, etc.) have shown [8,9,10,11,12] that for these biocomposites good mechanical strength characteristics can be obtained by means of appropriately selected agave fibers, with a compression molding process coupled with an optimized thermomechanical cure process. In accordance with these indications, the manufacture of the biocomposites was performed by mixing short fibers and green epoxy resin, in a special removable mold having dimensions 260 mm × 260 mm; the high compaction pressure was applied by using a 100-ton hydraulic press, Figure 1a. In detail, the compression molding lasted 24 h at a maximum pressure of 0.83 MPa [9].The cure process was integrated by a suitable thermal cycle, obtained by heating the mold to a temperature of 80 °C for about 2 h by means of an appropriate electrical resistances and monitoring the temperature using proper thermoresistances (see [9] for more details). Such a thermo-mechanical process has been used for all the materials considered in the present study.

The results of the literature point out that the best concentrations of biochar [33,47] in the epoxy resin is 2%, while random short-agave fiber biocomposites appear to achieve their best mechanical performance for 35 vf% of agave fibers [8,9,10,11,12,13]. Consequently, a biocomposite with an epoxy resin matrix that has 35 vf% of random short-agave fibers and 2 wt % of biochar should provide the optimal combination to improve the mechanical properties of the epoxy matrix. Biocomposite batches were manufactured mixing 2 wt % of biochar with the epoxy resin before adding the 35 vf% of agave fibers, this type of specimen was called BC2% and compared with batches produced with the same percentage of agave fibers but without biochar, called R+A. In order to confirm that even in the case of biocomposites with 35 vf% of short agave fibers the optimal quantity of biochar was always 2%, two other types of batches were produced: one with 1 wt % of biochar, called BC1%, and another with 4 wt %, called BC4%. In detail, four batches of each material (R+A, BC1%, BC2%, and BC4%) have been manufactured. From each batch 7 specimens have been cut, 3 for static loading and 4 for fatigue loading.

In detail, the initial impregnation of the fibers took place in excess of resin and the desired volumetric percentage of fibers, equal to 35 vf%, was obtained by adjusting properly the final thickness, equal to 3 mm, of the panel under pressure. Figure 1b illustrates the final phase of the pressing characterized by the leakage of the excess resin. Figure 1c–f show a panel of biocomposite with 35 vf% of agave fibers and 0, 1, 2, and 4 wt % of biochar extracted from the mould, before the necessary specimens are made for static and fatigue tensile tests. Finally, it is important to note that previous works [9] have demonstrated that the above mentioned optimal compression-molding process permits, in general, to control the main influence parameters, i.e., to obtain biocomposite panels with repeatable isotropic mechanical properties, corroborating also the randomly fiber distribution.

### 2.3. Material Testing

**Figure 2:**
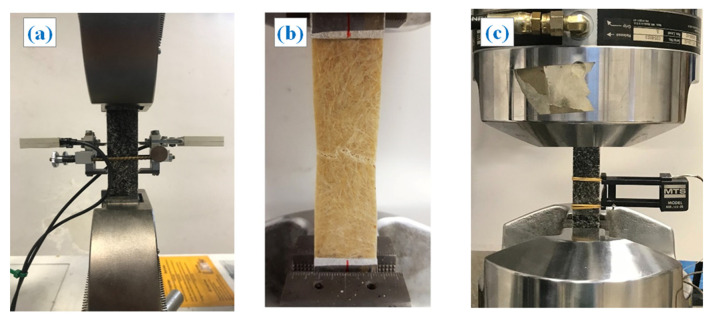
Testing of biocomposite specimens: (**a**) static tensile test specimen BC2%, (**b**) specimen R+A damaged at the end of the static tensile test, (**c**) fatigue test specimen BC2%.

In accordance with the ASTM D 3039/D 3039M-00 standard, the mechanical behavior of the different biocomposites under static loading were determined by tensile tests on rectangular specimens of 25 mm × 220 mm size. The tests were carried out on a Instron 3367 (static tests) with a traverse speed of 1 mm/min and on an MTS 810 type servo-hydraulic machine (fatigue tests). Figure 2a shows, as an example, the tensile test of a specimen with 2 wt % of biochar (BC2%), whereas Figure 2b illustrates a specimen without biochar (R+A), damaged at the end of the tensile test; finally Figure 2c shows a fatigue test on a specimen of BC2%.

## 3. Results

### 3.1. Biochar Characterization

**Figure 3:**
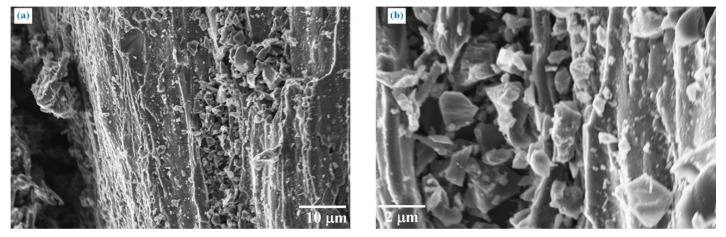
Biocarbon from pyrolysis of Mischantus with (**a**) 1000 and (**b**) 5000 magnifications, after ball milling.

Pristine biochar pellets were grinded using ball milling for 2 h to reduce the particle size, as shown in Figure 3.

**Figure 4:**
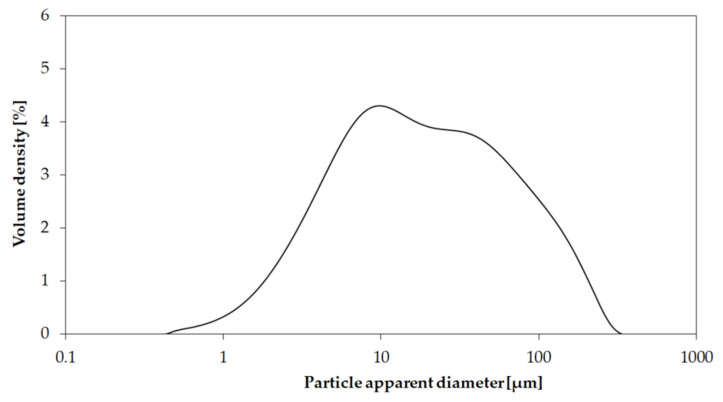
Particle size distribution of biocarbon from pyrolysis of Mischantus, after ball milling.

The biochar particles displayed mixed sizes with small particles with an average size of few microns on bigger particles that preserved the original channeled shape of Mischantus. FESEM observations were also proved by an analytical particle size distribution (Figure 4).

As reported by several authors [33,48], biochar particles with an average size up to hundreds of micron underwent to a disruption after dispersion in polymeric matrix by sonication. This leads to a uniform reduced particle size in composites.

**Figure 5:**
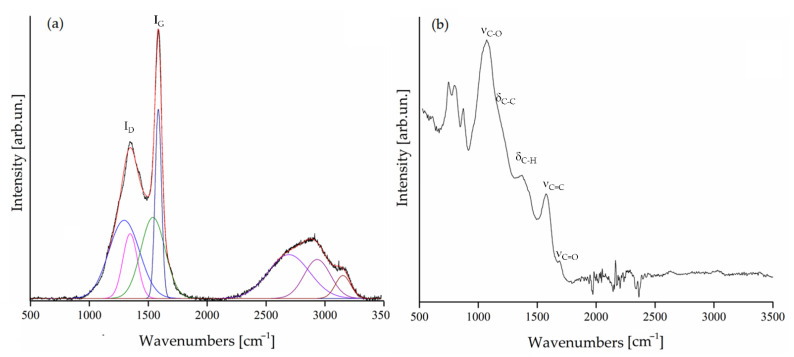
(**a**) Raman and (**b**) FT-IR ATR spectra of biochar in the region between 500 cm^−1^ to 3500 cm^−1^. Fitted Raman spectrum is reported in red, fitting component are reported as colored curves and original spectrum is reported in black.

Another relevant property of biochar is represented by the functionalities and graphitization that were investigated by Raman and IR spectroscopy as reported in Figure 5.

Raman spectrum (Figure 5a) showed an I_D_–I_G_ ratio of 1.2 proving the poor graphitization and highly disorder of the biochar. This was in good agreement with IR spectrum (Figure 5b) that showed a broad weak band around 1690 cm^−1^ (ν_C=O_) witnessing the presence of residual ketonic groups [49]. Furthermore, the bands of ν_C=C_ around 1580 cm^−1^ due to aromatic structures embedded in the carbonaceous matrix support the poor graphitization of the material.

### 3.2. Static Tensile Tests

**Figure 6:**
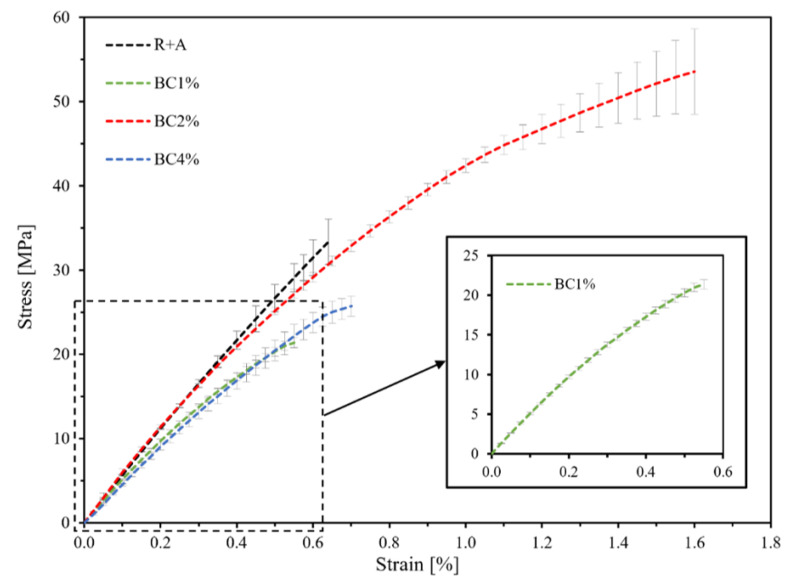
Tensile tests related to the analyzed biocomposites: (black) R+A, (green) BC1%, (red) BC2%, (blue) BC4%.

Figure 6 shows the static tensile curves relating to the specimens’ types R+A, BC1%, BC2%, and BC4%.

It is seen how the biocomposites with agave fibers but without biochar (R+A) exhibit a linear elastic behavior up to about 50% of the static failure load, followed by a subsequent stretch with decreasing stiffness until final failure. The biocomposites with agave fibers and 1 wt % of biochar (BC1%) instead show appreciable reduction of the failure stress *σ_u_* (about −38%); significant improvements in term of failure stress *σ_u_* (about +55%), failure strain *ε_u_* (about +250%) and specific failure energy *e_f_* (about +480%), are instead exhibited by the biocomposite with 2 wt % of biochar. Similarly to the biocomposite with 1 wt % of biochar (BC1%), the BC2% also shows an appreciable reduction in the failure stress *σ_u_* (about −28%).

Table 1 shows such results in terms of average values and standard deviation, along with the tensile Young’s modulus *E* provided by the same tensile tests.

From Table 1, it is possible to observe that passing from the biocomposite without biochar (R+A) to the toughened biocomposite with 2 wt % of biochar (BC2%), the most important improvement occurs in terms of failure strain that leads, as expected for a fiber reinforced composite, to an improvement of the ultimate stress, along with a significant improvement of the specific failure energy. Limited improvements are instead observed on the elastic modulus: the addition of 2 wt % of biochar to the matrix has resulted in Young’s modulus increment of about 5% (from about 5.37 to about 5.66 GPa). Biocomposite with 1 and 4% of biochar exhibit instead a failure strain comparable with that of the biocomposite without biochar; the specific failure energy and the Young modulus instead, are lower. Therefore, it is possible to state that the experimental analysis confirms how, as it has been observed [32], the addition of 2% of biochar leads to significant improvement of the mechanical characteristic, whereas the use of lower or higher concentrations lead to negligible improvements or appreciable reductions. By using low concentrations, biochar was merely a microsized structural defect in the epoxy resin reticule while at very high concentration prevent a proper reticulation inducing brittleness [30]. As mentioned in several papers, a woody biochar loading of 2 wt % magnify the polymeric matrix properties without compromising mechanical features due to well established interfacial interactions [33].

**Figure 7:**
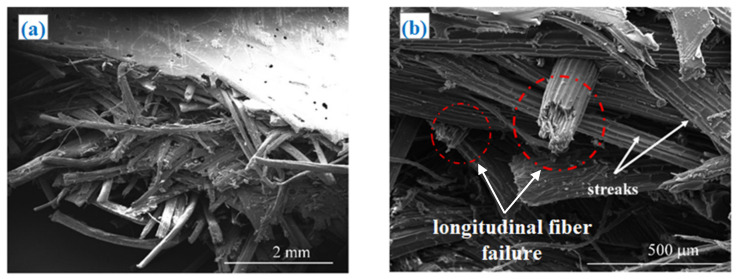
SEM micrographs of the fracture surfaces of the biocomposite R+A, subjected to static tensile loading with low (**a**) and high magnifications (**b**). Longitudinal fibers failure was red circled.

In order to better analyze the various damage mechanisms involved in the tensile tests, a Scanning Electron Microscopy (SEM) was used to analyze the fracture surfaces of the biocomposites studied; in brief, such an analysis has shown that the damage mechanisms of the biocomposites with biochar are similar to that of the biocomposite without biochar (see Figure 7 as an example of the R+A biocomposite).

In detail, from Figure 7 it is possible to observe the widespread “transversal failures” that starts in the zone where the fibers are not aligned with the load (see Figure 7a), mixed to longitudinal fracture of the fibers aligned to the loading direction (Figure 7b), and appreciable fiber splitting phenomena highlighted also by the streaks of the fiber surfaces (Figure 7b, red circled).

Moreover, the SEM micrographs analysis confirms that, also without biochar a sufficient fiber-matrix adhesion occurs and, consequently, it does not lead to significant pull-out phenomena: as it is seen in Figure 7b, in fact, the length of the free fiber segments is always less than 4 times the fiber diameter, i.e., always less than the critical fiber length.

Although the presence of biochar does not seem to modify the damage mechanism, it can significantly affect the matrix toughness and the fiber and matrix adhesion with a significant slowdown in the propagation speed of the microcracking of the matrix due to beneficial bridging effects.

### 3.3. Single-Fiber Pull-Out Tests

**Figure 8:**
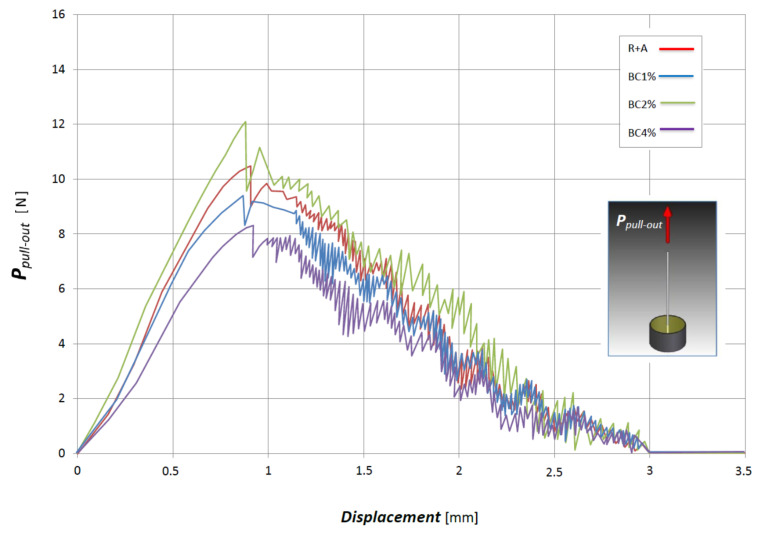
Pull-out tests on the various biocomposites analyzed (with sketch of the experimental setup).

In order to assess the actual effects of biochar in the fiber and matrix interface, a single fiber pull-out test has been performed on all the four biocomposites examined. In more detail, the pull-out tests have been carried out by using fibers having diameter of about 200 μm, embedded into small matrix cylinders having external diameter of 3 mm, with a matrix and fiber overlap length *l_e_* = 3 mm (see Figure 8). The experimental pull-out curves are synthetically reported in the same Figure 8.

Synthetically, Figure 8 shows that the addition of biochar influence significantly the pull-out strength, that appreciably increases for BC2% (about +20%), whereas it decreases for both BC1% (about −10%) and BC4% (about −20%), confirming that the optimal biochar fraction corresponds to 2%. It is important to note that, in accordance with the theory of the shear stress distribution that occurs at the fiber and matrix interface, exposed in [11], the improvement in the pull-out strength is strictly related to the effects of the biochar on stiffness (decreasing) and failure strain (increasing) of the green epoxy matrix. Obviously, such results confirm that the addition of 2% of biochar also improve the fiber and matrix adhesion under tensile loading.

### 3.4. Fatigue Tests

**Figure 9:**
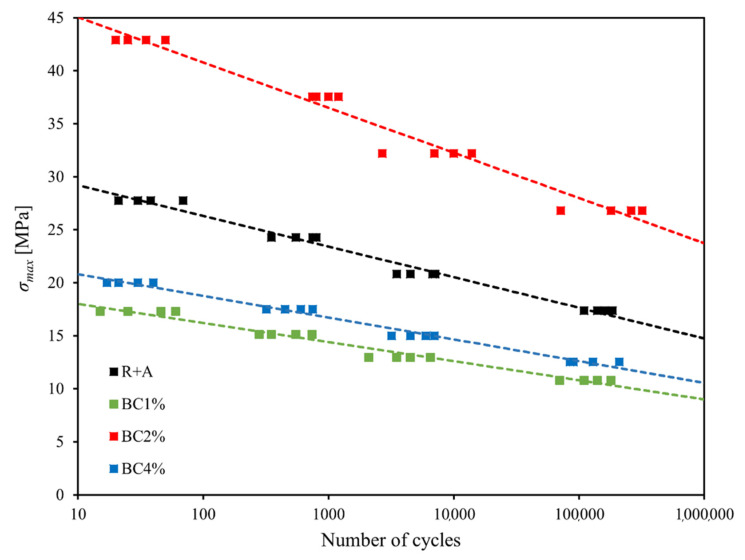
Results of fatigue tests performed on the different biocomposites analyzed.

In order to assess the actual effects of the biochar on the fatigue performance of biocomposites reinforced by agave fibers, all the different biocomposites above considered were subjected to systematic fatigue tests carried out by using the same servo-hydraulic machine MTS 810 used for static tests, with fatigue load ratio R = 0.1 (traction-traction fatigue), and a loading frequency of 5 Hz, which ensures, in both the examined cases, the absence of significant dissipative effects related to mechanical hysteresis; such tests have been performed in accordance with the ASTM D 3479/D 3479M-19 standard. The following Figure 9 summarizes the results of the fatigue tests for all the biocomposites analyzed, through the representation of the relative semi-logarithm Wohler curves.

In detail, the fatigue tests were carried out considering four distinct load levels (80, 70, 60, and 50% of the static failure load) and by using samples consisting of four specimens for each load level. The experimental points were linearly interpolated and reported in a classic semi-logarithmic diagram (Figure 6). For all the analyzed biocomposites, with and without biochar, the following Table 2 shows the numerical values of the results of the fatigue tests in terms of fatigue strength limit $\sigma_{F}$ at 10^6^ cycles, fatigue ratio φ = $\sigma_{F}$/$\sigma_{u}$ and relative percentage improvements.

From the analysis of the results of Figure 9 and Table 2, it is observed how, similarly to the static case, the fatigue strength of BC1% and BC4% decreases appreciably (−37 and −26%, respectively) compared to the R+A biocomposite; also the fatigue life decreases significantly (−3 and −2/−3 order of magnitude, respectively); synthetically, such percentage of biochar does not lead to any improvements of the fatigue performance.

On the contrary, the matrix toughening and the matrix and fiber adhesion improvement obtained with 2 wt % of biochar gives rise to a significant improvement in the fatigue performance of the analyzed biocomposite, quantitatively superior to the effect observed in static conditions. In particular, in terms of fatigue strength, a nearly constant increase is detected throughout the “high cycles fatigue” range (10^3^–10^6^ fatigue cycles); in detail, the addition of 2 wt % of biochar corresponds to an increase in fatigue strength of about 67%. In agreement with the fact that, in this case, the toughening effect of the biochar significantly slows down the speed of microcracking, that is in general the most important damage mechanism in the fatigue of composites, the most relevant improvements observed are in terms of fatigue life duration; in detail, from Figure 6 it is shown how passing from the biocomposite without biochar to that with 2 wt % of biochar, the fatigue life increases of at least 3 orders of magnitude. For example, for a 25 MPa fatigue load, the fatigue life goes from about 70 cycles for the biocomposite without biochar, to about 260,000 cycles for the biocomposite with 2 wt % biochar. Additionally, the appreciable increment of the fatigue ratio φ (from 0.40 to 0.43, see Table 2), which is indicative of the fatigue response of the material, confirms that the introduction of the biochar gives an actual improvement of the fatigue performance of the biocomposite, i.e., it is not the simple consequence of the static tensile strength increment. Furthermore, the comparison with the performance of the green epoxy resin alone shows the significance mechanical strength increments obtained by the biochar for both low and high cycles fatigue loading.

**Figure 10:**
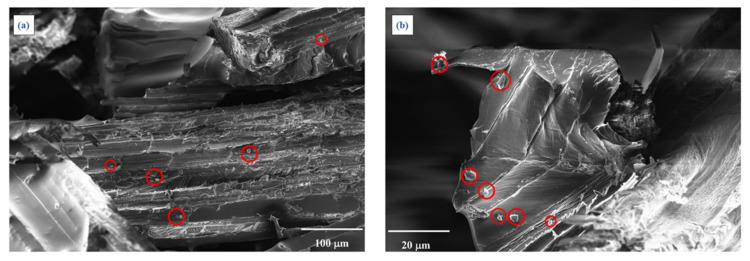
SEM micrographs of the fatigue fracture surfaces of a biocomposite specimen BC2%: (**a**) 15 and (**b**) 75 magnification; some biocarbon particles are highlighted by red circled.

The improving of fatigue behavior was reasonably imputable also to other interfacial effect of biochar. It acted as strengthening due to its highly dispersibility that promote the interaction between disordered its graphitic domain with polar fibers through π orbital or hydrolxilic residues interactions [50]. As shown in Figure 10, another interesting effect is represented by the distribution of biochar particles on the interphase between fibers and polymer bulk. This behavior promoted a better interaction between agave fibers and polymeric host with a decrement of delamination and a better fatigue performance.

Saba et al. [51] described in very systematically the effects of carbon filler addition to natural fibers epoxy composites enlighten the crucial role of interfacial interactions between fibers, fillers, and epoxy matrix. The improved interfacial interactions, promoted by carbon materials such as carbon black [52] or carbon nanotubes [53], simultaneously improved the fibers-resin adhesion and reduced the brittleness of the composites. We observed the same phenomena by dispersing the biochar proving the consistency of our approach with the observation previously reported for glass [54] and carbon [55] fibers.

## 4. Conclusions

A systematic experimental analysis of eco-friendly biocomposites properly manufactured by a green epoxy matrix reinforced with optimized short agave fibers and toughened by introducing suitable concentrations of biochar, has allowed to demonstrate that this material can be advantageously used to replace synthetical materials in structural applications characterized by significant static tensile or fatigue loading.

In detail, static tensile tests carried out on biocomposites with a fiber volume fraction Vf =35% and 2% of biochar gives rise to an improvement of the failure strain of about 160% with an increment of the tensile strength of about 55%, and of the specific failure energy of about 480%. There is also an appreciable increase in Young’s tensile modulus of about 5%. Besides the well-known matrix toughening, the introduction of 2% of biochar leads to an appreciable improvement of the matrix and fiber adhesion, how it has been demonstrated by proper single fiber pull-out tests. In terms of fatigue performance, the addition of 2 wt % of biochar increases the fatigue strength by about 67% and fatigue lifetime by least 3 orders of magnitude. Lower (1%) and higher (4%) weight fractions of biochar, instead, lead to reductions in static mechanical performances and unchanged fatigue performance.

The noticeable enhancement of the mechanical properties of the biocomposites with 2 wt % of biochar increases the possibility of using them in semi-structural and structural applications, especially in the automotive and naval sectors, characterized by the recurring presence of fatigue loading due respectively to random ground roughness and random wave height.

Unlike most of the chemical fiber treatments, these substantial enhancements are associated neither with an increase in the environmental impact nor with an increase in the cost of the biocomposite (that remain a low-cost material).

## Figures and Tables

**Table 1:** Results of tensile tests related to the various biocomposites analyzed.

Property	Symbol	Units	R+A	BC1%	BC2%	BC4%
Ultimate tensile stress	*σ_u_*	[MPa]	34.69	21.6	53.63	25.08
Standard deviation			±1.42	±0.55	±0.54	±0.34
Ultimate tensile strain	*ε* *_u_*	[%]	0.62	0.55	1.60	0.70
Specific failure energy	*e_f_*	[MJ/m^3^]	110 ± 6	65 ± 4	529 ± 21	100 ± 3
Young’s modulus	*E*	[GPa]	5.37	5.07	5.66	4.59
Standard deviation			±0.06	±0.13	±0.28	±0.12

**Table 2:** Results of fatigue tests related to all the analyzed biocomposites.

Material	Tensile Strength *σ_u_* (MPa)	Fatigue Limit *σ_F_* (MPa)	Fatigue Strength Increment	Fatigue Life Increment (Order of Magnitude)	Fatigue Ratio φ
R+A	34.69	13.87	-	-	0.40
BC1%	21.60	8.85	−37%	−3	0.41
BC2%	53.63	23.09	+67 %	+3	0.43
BC4%	25.80	10.28	−26%	−2/−3	0.41

## Data Availability

Not applicable.
